# ALMI—A Generic Active Learning System for Computational Object Classification in Marine Observation Images

**DOI:** 10.3390/s21041134

**Published:** 2021-02-06

**Authors:** Torben Möller, Tim W. Nattkemper

**Affiliations:** Biodata Mining Group, Bielefeld University, 33615 Bielefeld, Germany; tim.nattkemper@uni-bielefeld.de

**Keywords:** active learning, classification, deep learning, marine image annotation

## Abstract

In recent years, an increasing number of cabled Fixed Underwater Observatories (FUOs) have been deployed, many of them equipped with digital cameras recording high-resolution digital image time series for a given period. The manual extraction of quantitative information from these data regarding resident species is necessary to link the image time series information to data from other sensors but requires computational support to overcome the bottleneck problem in manual analysis. As a priori knowledge about the objects of interest in the images is almost never available, computational methods are required that are not dependent on the posterior availability of a large training data set of annotated images. In this paper, we propose a new strategy for collecting and using training data for machine learning-based observatory image interpretation much more efficiently. The method combines the training efficiency of a special active learning procedure with the advantages of deep learning feature representations. The method is tested on two highly disparate data sets. In our experiments, we can show that the proposed method ALMI achieves on one data set a classification accuracy A > 90% with less than N = 258 data samples and A > 80% after N = 150 iterations, i.e., training samples, on the other data set outperforming the reference method regarding accuracy and training data required.

## 1. Introduction

The human impact on the marine ecosystem has increased in recent decades [1]. Activities that have a major impact include oil drilling, fishing, and wind turbine deployment. An important factor in monitoring marine biodiversity and maintaining sustainable fish stocks is marine imaging [2,3]. Possible applications are, for example, creating a time series of different species by detecting and classifying species in the images. However, computational support is needed to make the best use of the vast amounts of data generated, e.g., by stationary underwater observatories [4] or seafloor observation systems [5]. A lot of work exists in the context of (semi-)automated detection and classification of species in marine images [3,6,7,8,9,10,11,12]. All these works employ some kind of machine learning algorithm to render a data-driven model of the task to be performed (like object detection or classification). Such a machine learning approach towards (semi-)automatic image interpretation requires a training set of images (or image patches) and expert annotations, usually represented as (taxonomic) labels associated with the images collected with some image annotation software such as BIIGLE 2.0 [13]. In almost all works published, these images are in fact image patches, marked by domain experts in large images showing an underwater scenery containing multiple objects. The detection and extraction of these patches showing single objects can be done by experts, sometimes supported by computational methods that often employ unsupervised learning [14,15,16] or even citizen scientists [17]. However, one task that cannot be supported straightforwardly with computational methods or non-experts is the final classification of objects to taxonomic categories or morphotypes, and this task is addressed in this work.

One main problem in providing computational support for taxonomic classification by employing supervised learning classifiers is the amount of manual expert work required to collect a training set of labeled image patches of sufficient size for all object classes. To collect such a set, several challenges must be faced, some of them very special for underwater computer vision applications:

1. Limited background knowledge: Often, it is not known a priori which species can occur in the data set.
2. Expensive expert annotation: Quality controlled annotations are expensive in the context of marine imaging because expert knowledge from the domain of marine biology is needed for the annotation process.
3. Low abundant classes: It is time-consuming to manually find a sufficient number of examples of rare species for the training set.

One approach that is particularly well suited to tackling these three challenges is active learning. The core idea of active learning is to select training samples automatically from the set of samples to be classified instead of leaving the selection of training samples to human experts. This automated selection of training samples is usually done in an iterative fashion. First, a training sample is selected automatically from the set of all samples to be classified. Next, the selected sample is labeled by an expert, and the process continues by going back to the first step. This is repeated until enough training samples have been selected and labeled. Performing the first step—selecting a training sample—requires an explicit description of a so-called *sampling strategy*.

In light of the three challenges listed above, we define the following criteria for an efficient sampling strategy in this context. In order to make efficient use of the data and domain expert’s time collecting a training set, the sampling strategy should…

a. *…* avoid samples that do not show any instance of a class (i.e., species),
b. *…* prioritize samples that show an instance of a class that is not yet in the training set,
c. *…* prioritize samples that show an instance of a class that is underrepresented in the training set, and
d. *…* prioritize samples that can help to discriminate a class better from the other classes.

A number of works have been published in the field of active learning in recent years [7,18,19], and one popular method is active learning using uncertainty sampling [18]. The basis of uncertainty sampling is the estimation of a classifier’s uncertainty regarding the classification of each sample. This allows the automatic selection of the sample with the highest estimated uncertainty to be labeled by the experts in a training step to increase the potential of a classifier’s ability to discriminate the according class from the other classes. In [19], the authors propose a two-class active learning method that generates a clustering prior to the actual classification. Then, the algorithm assigns a higher priority to examples the closer they are to the classification boundary and the closer they are to a cluster centroid. In [7], an initial clustering is also performed, and relevance scores are assigned to the clusters. The relevance score is supposed to represent the extent to which the cluster can obtain samples that are likely to have greater potential to enhance the classifier’s performance. The relevance scores are then used to determine a cluster from which a sample is randomly drawn. The method has been shown to perform very well on a marine image data set. However, the method employs so called hand crafted feature representations to classify the images, i.e., so-called *dominant color features*, an established color feature representation method that is often applied in image retrieval contexts. The dominant color feature for an image patch is extracted by grouping the pixels (i.e., their rgb-colors) into a number of five clusters using the modified median cut algorithm [20]. The mean of all color vectors in the cluster containing the highest number of color vectors is the image patch’s dominant color feature. Dominant color features are not expected to work well on other datasets containing species that are visually distinguished by shape rather than color. This is likely to be the case for datasets from a different location, or perhaps even a dataset taken at the same location but with a new hardware setup. In recent years, convolutional neural networks have been successfully proposed as a very powerful approach to computer vision problems, making the selection and tuning of classic hand crafted features like dominant colors obsolete.

In this paper, we propose ALMI, a new active learning method for the object classification in marine images using a generic deep learning feature representation. ALMI takes sub-images, referred to as image patches in the following, and returns for each patch a class label, describing its content. ALMI is built on two conceptual ideas: First, it combines uncertainty sampling with relevance sampling to automatically select the next sample to be classified by domain experts and added to the training set. Second, it achieves a new level of flexibility by employing deep learning features instead of hand-crafted features like dominant colors (see above) proposed in prior works.

We use two different data sets from marine imaging to demonstrate our method and evaluate its performance in comparison with other methods. The two data sets differ regarding the location and water depth where they were recorded, and consequently regarding the taxonomic composition of species they contain. Moreover, the images from one data set were taken from a cabled fixed underwater observatory (FUO) while the images from the other data set were taken from a moving towed ocean floor observation system (OFOS). Our experiments show that our method is able to perform well on image sets that differ in various aspects. In both data sets, our generic approach outperforms the state-of-the-art methods without any extensive tuning towards the individual data set.

The data sets used as input for ALMI in our experiments are explained in more detail in the next section. Section 3 describes the proposed method ALMI, and the results of the evaluation are given in Section 4. Section 5 will discuss the evaluation and wrap our findings up.

## 2. Materials

**Figure 1:**
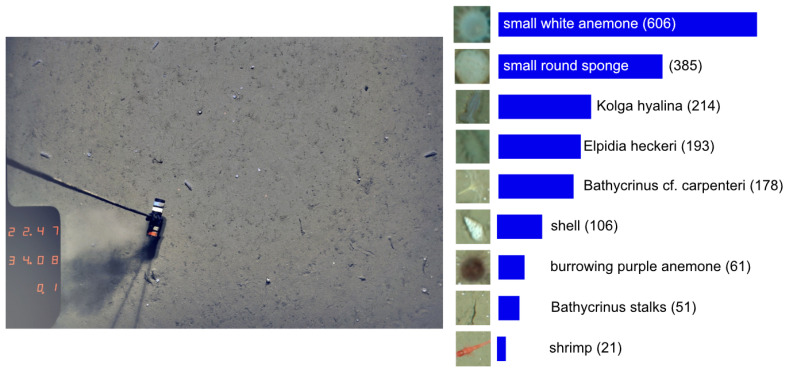
Images from the Hausgarten observatory (HG). **Left**: An original image. **Right**: The Hausgarten data set (HG) as used in experiments in Section 4.1. The numbers in brackets indicate the number of samples in a class. IMAGE: AWI OFOS team.

The first data set (see Figure 1) was created from an open-access, still image data set taken at the Hausgarten observatory with an Ocean Floor Observation System (OFOS) [21,22]. The original images are publicly available as described in the Data Availability Statement at the end of this paper. The Hausgarten observatory currently includes 21 stations located between (N 78.5°, E 05°) and (N 80°, E 11°) between Greenland and Svalbard. The OFOS was towed to a research vessel and took images of size 3504×2336 at a depth of 2500 m. From the OFOS images, sub-images showing one object (like a sea star or a crustacean for instance) were extracted by the authors of [6]. The resulting data set will be referred to as Hausgarten dataset (HG). The dataset HG was used in [6] to evaluate the COATL learning architecture and will be used in our experiments in Section 4.1. The HG data set used in this work contains 1815 image patches grouped into 9 classes.

**Figure 2:**
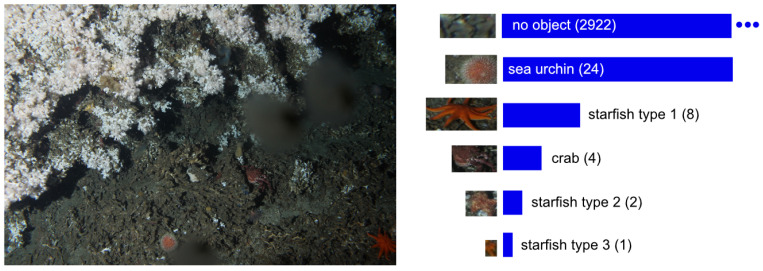
Images from the LoVe observatory. **Left**: An original image. **Right**: The LoVe data set (LV) as used in the experiments in Section 4.2. The numbers in brackets indicate the number of samples in a class. The three dots indicate that the bar for the images containing no object is out of scale for better visualization.

The second data set (see one example image in Figure 2) was created using images from the Lofoten-Vesterålen (LoVe) Ocean Observatory. The original images are publicly available as described in the Data Availability Statement at the end of this paper. LoVe is a cabled fixed underwater observatory located at (N 68° 54.474′, E 15° 23.145′) in the Norwegian Sea about 22 km offshore. The observatory monitors a coral reef at a depth of about 260 m. Among other sensors, the observatory is equipped with a high-resolution digital camera taking images of the coral reef. One image of size 5184×3456 pixels is taken once per hour. The change detection method proposed in [16] was used to extract sub-images containing at most one object per sub-image from 24 LoVe images in an unsupervised fashion. The resulting dataset will be referred to as LoVe data set (LV). The LoVe dataset was used in [7] to evaluate an active learning method and will be used in our experiments in Section 4.2. The LV dataset used in this work contains 3031 image patches grouped into 6 classes. It mainly consists of one image patch class “no object” showing no objects of interest (see Figure 2 on the right).

## 3. Methods

**Figure 3:**
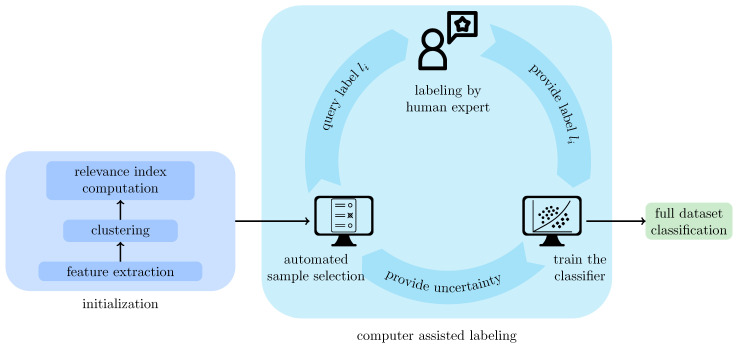
Overview of the active learning method. In preparation for the selection of the training samples, the samples are clustered, and a relevance index is assigned to each cluster. Furthermore, a feature vector is extracted for every image. For the selection of the training images, three consecutive steps are repeated iteratively: (i) A training sample is chosen from the set of images according to the sampling strategy and the state of the classifier. (ii) The sample is labeled by an expert. (iii) The classifier is updated. The trained classifier can then be used to classify the remaining samples.

The proposed active learning workflow ALMI (see Figure 3) takes a set $I=\left\{I_{i}\;|\;1\leq i\leq N\right\}$ of images $I_{i}$ (from here on, we will use the term image instead of image patch) as input and assigns the images to classes that have a semantic meaning. First, a fully automatic initialization step is performed to prepare the data for the semi-automatic labeling process where semantic classes are found and training samples of all classes are labeled and added to the training set. The initialization step starts by extracting from each image $I_{i}$, a feature vector $f_{i}$ that represents the image in a lower-dimensional (here 300 dimensional) vector space. Next, the feature vectors are grouped into *M* clusters $C_{j},\left(1\leq j\leq M\right)$ where features that are similar to each other belong to the same cluster. For 1≤i≤N, we denote by $c_{i}$ the unique cluster index *j* with $f_{i}\in C_{j}$. Moreover, a relevance score is computed for each cluster. This score estimates the potential of the cluster’s items to improve the classifier’s learning performance in learning new classes not represented in the training set (see Section 3.2 below).

Next, the training set is composed, and in each iteration, the following three steps are performed:

1. A sample image $I_{i}$ is chosen automatically according to the sampling efficiency criterion (defined below in the Sampling efficiency algorithm section)
2. An expert classifies the sample into a class found in a previous iteration or into a new class.
3. The classifier is retrained to update the uncertainties used in the sampling criterion (step 1).

The trained classifier can then be used to classify the remaining samples.

### 3.1. Feature Extraction

For further processing, for each image $I_{i}$, a feature vector $f_{i}$ is computed that describes the image in a lower dimensional vector space. Due to the limited background knowledge problem formulated in the introduction, the image feature representation cannot be built with hand-crafted features using heuristics without a strong loss in generalization (see Section 1). Instead, we propose to use the InceptionV3 Net [23], a fully convolutional deep learning network, to extract features $f_{i}$ for any network input $I_{i}$. These features however are abstract and are learned automatically during a pre-training step (see below).

**Figure 4:**
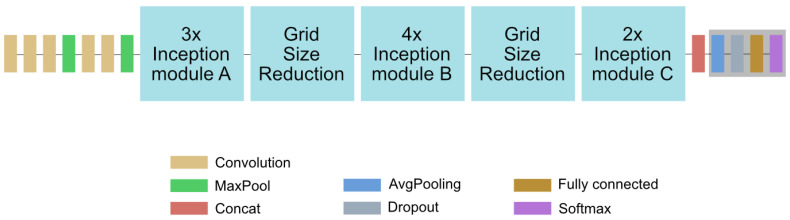
The InceptionV3 Net: Most layers of InceptionV3 are organized in so-called inception modules (see [23]). These inception modules compute multiple convolutions that are concatenated and used as the output of the module. The output of the last inception module before the classification layers (shaded gray) is used for feature extraction in our workflow.

A deep learning network takes the image $I_{i}$ as input and passes it through a number of so-called *layers* that transform the input and pass it to the next layer until the the image is classified in the last layer. In the case of fully convolutional networks like InceptionV3, the layers mainly consist of a number of filters. In the InceptionV3 Net (see Figure 4), the layers are grouped into so-called inception modules inspired by the Inception Net described in [24]. The inception modules take the output of the previous inception module as input (or the original image in case of the first inception module) and perform multiple convolutions with different kernel sizes. The convolution results are then stacked on top of each other and passed to the next inception module. The output of the last inception module can be seen as a feature vector that is passed to the last layer for classification The filter-weights are not predefined by human experts but are learned during the training, where images are classified and the filter-weights are adjusted in an iterative process to optimize the classification performance. In case of the InceptionV3 Net about $25\times 10^{6}$ parameters (mainly filter-weights) are learned during the training.

To make sure that the filter-weights are set properly, we use an InceptionV3 Net that was pretrained on a large set of images, the ImageNet [25]. ImageNet is a list of web images that provides access to more than $14\times 10^{6}$ annotated and quality-controlled web images. The list includes images showing examples of a variety of concepts such as sports, foot, animal, fish, etc. The subset of marine animals contains 1348 images. The InceptionV3 Net pre-trained on the ImageNet data set used in this work was downloaded using tensorflow [26].

To generate a feature vector $f_{i}$ for one images $I_{i}$, the images are fed into the pretrained InceptionV3 Net and propagated through the layers. The output of the last layer of the last inception module will be denoted by $\tilde{f_{i}}$. As these features $\tilde{f_{i}}$ are 2048-dimensional, we reduce the dimension in order to enhance the computation time and performance of the classifier. To do so, we use Principal Component Analysis (PCA) [27]. PCA is a method for dimension reduction that can be thought of as understanding the feature vectors as datapoint in the 2048-dimensional euclidean space and transforming them into a new coordinate system that is determined in the following way. The first axis is determined to minimize the sum of the squared distances between itself and each data point. The other axes are determined one by one in the way that each axis minimizes the sum of squared distances between itself and the data points under the condition of being orthogonal to all previously determined axes. After transforming the feature vectors in this way, all but the first 300 coordinates of each feature vector can be omitted without loosing too much information (compare the explained variance in Section 4.1 and Section 4.2). The feature vectors obtained this way will be denoted by $f_{i}$.

### 3.2. Cluster Relevance

The feature vectors are grouped into *M* clusters (i.e., groups of feature vectors that are similar to one another) using a cluster method that takes the feature vectors as input and returns for each feature vector $f_{i}$ a cluster index $c_{i}$ ($1\leq c_{i}\leq M$) as output. The choice of the clustering method is not crucial in this context and is in general not dependent on the data set or the kind of imaging setting. The method we use for the dataset HG is agglomerative clustering. Agglomerative clustering starts with *N* clusters and assigns each feature vector to its own cluster. Next, the number of clusters is reduced by iteratively choosing two clusters according to a given criterion (here: wards criterion) and merge them until only *m* clusters remain. Here, we use wards criterion that chooses the two clusters to be merged in the way that the increase of the in-cluster variance is minimal.

In the case of the dataset LV, the images $I_{i}$ were extracted automatically from larger images using the change detection method BFCD [16] which only is applicable to images from a fixed camera. In its core, BDFC extracts the images $I_{i}$ containing maximum one object per image as sub-images from a time-series of large scale images. This is done by clustering the pixel-wise differences of the large scale images to the pixels of the mean image of the large scale images. In that process not only the images $I_{i}$ are returned but also a cluster index $c_{i}$ (1≤i≤M) for each image is returned, so no additional clustering is required for the dataset LV.

For both data sets, the relevance scores of the clusters are computed as follows. For a cluster *j* (1≤j≤M), let

(1) $$S_{j}=\left\{f_{i}\;|\;1\leq i\leq N\;\wedge \;c_{i}=j\right\}$$

denote the set of feature vectors that belong to cluster *j* and let

(2) $$\;m_{j}=\frac{1}{\left|S_{j}\right|}\cdot \sum_{f_{i}\in S_{j}}f_{i}$$

denote the centroid of cluster *j*. With the mean of all feature vectors

(3) $$C=\frac{1}{N}\cdot \sum_{i=1}^{N}f_{i}$$

and the Euclidean distance $d_{2}$, the relevance score $r_{j}$ of cluster *j* is defined as the distance

(4) $$r_{j}=d_{2}\left(C,m_{j}\right)$$

between the centroid of cluster *j* and the mean of all feature vectors.

### 3.3. Sampling Efficiency Algorithm

Motivated by the criteria (a–d) defined in the introduction, we implement a sampling algorithm to select the next training sample in two steps:

Step 1 —Cluster selection: For selecting, a cluster, let the activity score $a_{j}$ denote the number of times a sample has been selected from cluster *j* in the previous iterations. By defining
  (5) $$x_{j}=\left\{\begin{array}{cc} \infty & \mathrm{if}\;a_{j}=0\\ \frac{r_{j}}{a_{j}} & \mathrm{else} \end{array}\right.$$
  and selecting the cluster
  (6) $$\tilde{j}=\underset{j}{arg max}\left(x_{j}\right)$$
  it is ensured that the frequency that a sample is drawn from cluster *j* is approximately proportional to $r_{j}$.
Step 2 —Training sample selection: Let $\left\{\omega_{1},\ldots ,\omega_{K^{\left(T\right)}}\right\}$ denote the $K^{\left(T\right)}$ classes that are present in the training set during the sample selection in iteration *t*. If $K^{\left(T\right)}\geq 2$, uncertainty sampling [18] is used to draw a sample from the cluster $\tilde{j}$ selected in step 1: Given a sample $f_{i}$ to be classified, a chosen classifier (e.g., the support vector machine (SVM) [28] that is used in the experiments in this paper) computes for each class $\omega_{k}$ the probability $p_{i,k}$$\left(1\leq k\leq K^{\left(T\right)}\right)$ that $f_{i}$ belongs to $\omega_{k}$. With
  (7) $$\delta_{i}^{\left(t\right)}=\left\{\begin{array}{cc} 1 & \mathrm{if}\;\mathrm{sample}\;i\;\mathrm{has}\;\mathrm{been}\;\mathrm{labeled}\;\mathrm{before}\;\mathrm{iteration}\;t\\ 0 & \mathrm{else} \end{array}\right.$$
  the characteristic function that indicates if a sample has been labeled in an iteration $t^{\prime }<t$, the uncertainty of the classifier regarding the classification of a feature $f_{i}$ can then be expressed as
  (8) $$u_{i}=\left\{\begin{array}{cc} 0 & \delta_{i}^{\left(t\right)}=1\\ 1-\max_{1\leq k\leq K^{\left(T\right)}}\left(p_{i,k}\right) & \mathrm{else} \end{array}\right.$$
  To select the sample where the classifier is most uncertain, the sample $f_{\tilde{i}}$ with
  (9) $$\tilde{i}=\underset{\left\{1\leq i\leq N\;|\;c_{i}=\tilde{j}\right\}}{arg max}u_{i}$$
  is selected. In case $K^{\left(T\right)}<2$, a classifier can not be trained and a sample is drawn randomly with uniform distribution from the cluster $\tilde{j}$ selected in step 1.

### 3.4. Classification Uncertainty

As a last step in each iteration, the classifier has to be trained to obtain the uncertainties that are used in the next iteration. In the *t*-th iteration, a number of *t* samples have been labeled by the expert. The labels of the labeled samples are propagated to the remaining samples using the clusters found in Section 3.2. To each cluster *j*, the label ${\hat{l}}_{j}$ is assigned that occurs most often in cluster *j*, according to

(10) $${\hat{l}}_{j}=\underset{1\leq k\leq K^{\left(T\right)}}{arg max}\left(\left\{1\leq i\leq N\;|\;\delta_{i}^{\left(t\right)}=1\;\wedge \;c_{i}=j\;\wedge \;l_{i}=\omega_{k}\right\}\right)$$

The labels assigned to the clusters are then used to assign a label to each sample, according to

(11) $${\tilde{l}}_{i}=\left\{\begin{array}{cc} l_{i} & \mathrm{if}\;\delta_{i}^{\left(t\right)}=1\\ {\hat{l}}_{c_{i}} & \mathrm{else} \end{array}\right.$$

The features $f_{i}$ and their labels ${\tilde{l}}_{i}$ are then used to train the classifier. The trained classifier is then used to predict for each sample $f_{i}$ and each class $\omega_{k}$ the probability $p_{i,k}$$\left(1\leq j\leq K^{\left(T\right)}\right)$ that $f_{i}$ belongs to $\omega_{k}$. These probabilities are then used in the next iteration to compute the uncertainties during the selection of the next sample.

## 4. Evaluation

ALMI is evaluated on the LoVe dataset and the Hausgarten dataset. The real-life application with the human expert iteratively labeling the data as described above is simulated with the data sets LV and HG that have been entirely labeled with gold standard classifications $g_{i}$ by domain experts in advance. During each iteration, when the label for an image $I_{i}$ is queried, the a priori determined label $g_{i}$ is assigned to $I_{i}$. As a classifier, the Support Vector Machine [28] (SVM) is used. The main idea of the SVM for two classes is to find a hypersurface that separates the classes in the training set. To do so, the SVM transform the samples into a higher-dimensional vector space until a separating hyperplane can be found. The samples and the hyperplane are then transformed back to the original vector space ending up with a hypersurface that separates the training data. A new sample can then be classified by determining on which side of the hypersurface the sample is located.

For each of the data sets, published results of a state-of-the-art method are available for comparison. Each of these methods adds the training samples one by one in an iterative fashion to the training data set similar to the iterations of the computer-assisted labeling described in Section 3.3 and Section 3.4. At the end of each iteration *t*, the classification performance is evaluated on a test set $T=\left\{I_{i_{1}},\ldots ,I_{i_{\hat{N}}}\right\}\subset I$ with the number $\hat{N}$ of test samples as described in the following two subsections.

To compute a classifier’s performance on T, let $g_{i_{\tau }}$ denote the gold standard label assigned to image $I_{i_{\tau }}\in T$ by human experts. Furthermore, let $h_{i_{\tau }}^{\left(t\right)}$ denote the label assigned to feature $f_{i_{\tau }}$ by the classifier when trained with *t* training samples. An often-used method to evaluate a classifier’s performance is to compute the accuracy defined by

(12) $$a^{\left(t\right)}=\frac{1}{\left|T\right|}\left|\left\{1\leq \tau \leq \hat{N}\;|\;h_{i_{\tau }}^{\left(t\right)}=g_{i_{\tau }}\right\}\right|$$

which describes the proportion of correctly classified samples in all classified samples. However, for a fair evaluation of the methods on the LoVe dataset and the Hausgarten dataset this performance measure will be changed slightly to match the evaluation in [6] and [7] as described in the following two subsections.

### 4.1. Evaluation on the Dataset HG

In this experiment, the proposed method is evaluated on the dataset HG. First, the result of the principal component analysis is inspected. As described in Section 3.1, the PCA is used to reduce the InceptionV3 Net features from a length of 2048 to a length of 300. For this dataset, the explained variance of the first 300 principal components was determined to be 94.2%. Next, the proposed method is compared to the *COATL*-approach proposed in [6]. The core idea of COATL is to use different classifiers depending on the number of available expert labels. No classifications are made until five labels are available. From five to 20 available labels, a K-Nearest-Neighbors approach is used. From 20 to 400 labels, an SVM is used. From 400 to 1500 labels, an H2SOL [6] is used. Moreover, when more than 1500 labels are available, a convolutional neural network is used.

As proposed in [6], after each iteration *t*, the performance is evaluated on all samples except for the *t* labeled samples. By doing so, the test data set consists of N−t images after iteration *t*. To avoid testing on a too small test dataset, only 1500 iterations are performed which leaves 315 test samples after the last iteration. In both methods COATL and ALMI, a number of $n_{k}$ first classifications are neglected. In case of COATL, this number is set to $n_{k}=5$. In case of ALMI, all image classifications are neglected before more than one class has been learned, i.e., $K^{\left(T\right)}>1$ with $K^{\left(T\right)}$ as the number of classes learned after *T* iterations. That is why a slight modification of the accuracy given in Equation (12) is used in this experiment.

(13) $$a_{HG}^{t}=\left\{\begin{array}{cc} \frac{1}{N-t}\times \left|\left\{1\leq i\leq N\;|\;h_{i}^{\left(t\right)}=g_{i}\;\wedge \;\delta^{\left(t\right)}=0\right\}\right| & \mathrm{if}\;t\geq 5\;\wedge \;K^{\left(T\right)}\geq 2\\ 0 & \mathrm{else} \end{array}\right.$$

**Figure 5:**
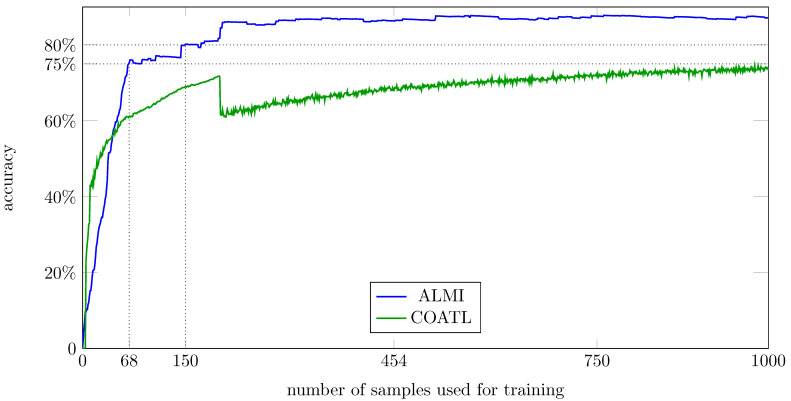
The accuracy values according to Equation (13) for ALMI (blue) and COATL (green) achieved on the Hausgarten dataset. The vertical straight lines show how many labels are required to achieve a performance of 75% resp. 80%.

The accuracy $a_{HG}^{t}$ is computed for the proposed method and for COATL after each iteration. That is done 10 times for each method, and the average accuracies for each method and each number of samples *t* are shown in Figure 5.

The results show that our new proposed generic method shows a steeper learning rate than COATL, even without any particular tuning for this data. To achieve an accuracy of 75%, the proposed method just needs 68 labels (4.5% of the training data), while COATL needs 1023 labels (68.2% of the training data). An accuracy of 80% is achieved by the proposed method with 150 labels (10% of the training data), while COATL does not achieve an accuracy of 80%.

**Figure 6:**
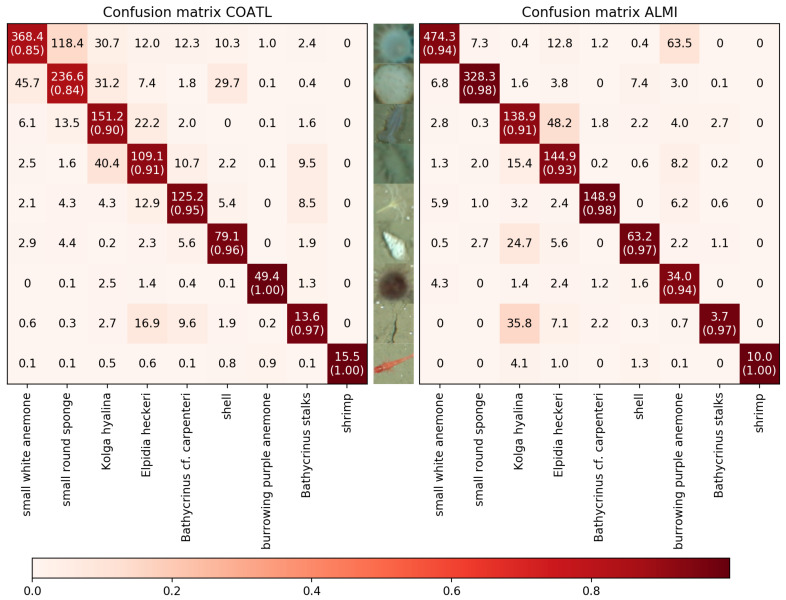
Left: confusion matrix of COATL after 150 iterations. Right: confusion matrix of ALMI after 150 iterations. The ι-th row of a matrix shows in the κ-th column how often an instance of class ι has been labeled as κ. The number in brackets in a cell (ι,ι) represent the class-wise accuracy obtained by the classifier for class ι according to Equation (14). The color of a cell (ι,ι) encodes the class-wise accuracy of class ι according to the color bar on the bottom. The colors in the other cells (ι,κ) represent the fraction of the number in (ι,κ) in the sum of all numbers in the ι-th row or κ-th cell.

After 150 iterations when ALMI has an accuracy of 80.0%, the accuracy of COATL is 69.9%. To show which species are effected, the confusion matrix of COATL and ALMI after 150 iterations is shown in Figure 6.

The rows represent the true labels while the columns represent the predicted labels, i.e., the number in row ι column κ represents how often an instance of class ι has been predicted as class κ in average over the 10 runs. The numbers in brackets on the main diagonal show the class-wise accuracies and have been computed as follows. For a class ι let

- $TP_{\iota }$ denote the number of instances of class ι that have been correctly classified as class ι,
- $TN_{\iota }$ denote the number of instances of any class κ≠ι that have not been classified as class ι,
- $FP_{\iota }$ denote the number of instances of any class κ≠ι that have incorrectly been classified as class ι, and
- $FN_{\iota }$ denote the number of instances of class ι that have been incorrectly classified as class κ≠ι.

The accuracy of class ι is then defined as

(14) $$a\left(\iota \right)=\frac{TP_{\iota }+TN_{\iota }}{TP_{\iota }+TN_{\iota }+FP_{\iota }+FN_{\iota }}$$

On the first glance, the class-wise accuracies obtained by the proposed method ALMI seem to be better or equal to the class-wise accuracies obtained by COATL except for the class “burrowing purple anemone”. In fact, also for the class “shrimp” COATL performs a little better, as ALMI has more false negatives than COATL. That is not reflected by the accuracy as the number of true negatives is quite high compared to the number of false negatives for this underrepresented class leading to a accuracy of >0.95 for both methods.

As the overall accuracy shows, ALMI still outperforms COATL. As can be seen in the upper right triangles of the confusion matrices, this is mainly due to fact that ALMI fixes the problem that COATL tends to assign species incorrectly to more abundant classes.

### 4.2. Evaluation on the Data Set LV

In this experiment, ALMI is evaluated on the dataset LV. First, the feature vectors are inspected. As described in Section 3.1, the InceptionV3 Net features are reduced from a length of 2048 to a length of 300 using principal component analysis. For this dataset, the explained variance of the first 300 principal components was determined to be 95.6%.

Next, ALMI is compared to the active learning approach based on dominant color features, described in [7]. As in [7], 200 runs of the experiment have been conducted. Both ALMI and the method described in [7] do not classify any sample before the number *K* of classes in the training set exceeds 1. Several data-specific aspects had to be considered in this evaluation. First, the test dataset is not strictly separated from the training dataset and the classification performance is evaluated after each iteration on the whole dataset including the labeled training data. Second, the expert labels available after *t* iterations are included in the evaluation. To do so, let for 1≤i≤N

(15) $${\hat{h}}_{i}^{\left(t\right)}=\left\{\begin{array}{cc} l_{i} & \mathrm{if}\;\delta_{i}=1\\ h_{i}^{\left(t\right)} & \mathrm{else} \end{array}\right.$$

denote the labels that are used to train the classifier.

Third, to take the strong data imbalance and the dominating abundance of images with no objects (see Figure 2 top right) into account, the performance measurement had to be adapted. Otherwise, the performance measurement would easily measure very high accuracies even for a naive classifier, classifying all images to the no object class. Thus, to neglect the correctly classified no object-samples in the HG experiments the following changes were applied to the number of images considered in the evaluation

(16) $$\hat{N}=\left|\left\{1\leq i\leq N\;|\;\neg \left({\hat{h}}_{i}^{\left(t\right)}=0\;\wedge \;g_{i}=0\right)\right\}\right|$$

and to the performance measure

(17) $$a_{LV}^{t,r}=\left\{\begin{array}{cc} \frac{1}{\hat{N}}\times \left|\left\{1\leq i\leq N\;|\;{\hat{h}}_{i}^{t}=g_{i}\;\wedge \;{\hat{h}}_{i}^{t}\neq 0\right\}\right| & \mathrm{if}\;K^{\left(T\right)}\geq 2\\ 0 & \mathrm{else} \end{array}\right.$$

**Figure 7:**
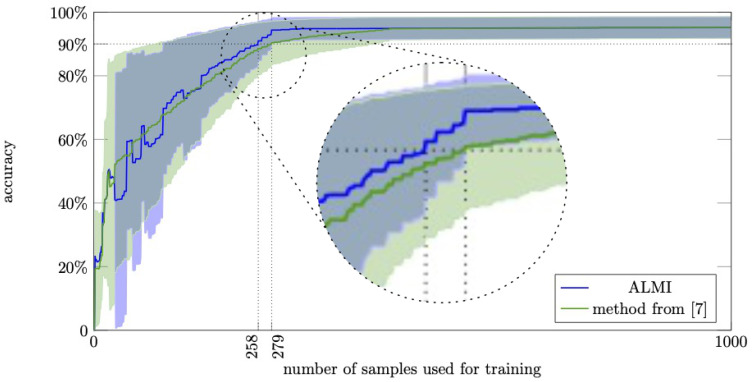
The accuracy values according to Equation (17) for ALMI (blue) and the method proposed in [7] (green) achieved on the dataset LV. The blue and green areas visualize the standard deviation as computed in Equation (19). The circle shows a magnification of the plots in the region around the 90% accuracy mark. The vertical straight lines show how many labels are required to achieve a performance of 90%.

The accuracy $a_{LV}^{t,r}$ is computed for ALMI and COATL after each iteration *t*. The superscripts *t* and *r* denote here that the accuracy $a_{LV}^{t,r}$ has been measured in the *r*-th run of the experiment in iteration *t* (i.e., with *t* labeled samples). Along with the mean, Figure 7 shows the standard deviations computed and visualized as follows. With

(18) $$\mu^{t}=\frac{1}{200}\sum_{r=0}^{200}a_{LV}^{t,r}$$

denoting the mean of the accuracy at iteration *t* averaged over all runs of the experiment, the standard deviation of the accuracies after iteration *t* is defined as

(19) $$\sigma^{t}=\sqrt{\sum_{r=0}^{200}{\left(\mu^{t}-a_{LV}^{t,r}\right)}^{2}}$$

The area between the curves of $\mu^{t}-\sigma^{t}$ and $\mu^{t}+\sigma^{t}$ is then filled with a semi-transparent color.

Figure 7 shows that also on this dataset the results of ALMI outperform previously published results on the same dataset. To achieve an accuracy of 90%, ALMI needs 258 labels (8.5% of the dataset), while the method in [7] needs 279 labels (9.2% of the dataset). When the method in [7] achieves 90% accuracy, ALMI has already reached 94% accuracy. In other words, ALMI has about 40% fewer misclassifications when trained with 279 labels. Regarding the standard deviation, the plot shows two things. First, after about 110 iterations, the accuracies achieved by ALMI vary less than the accuracies achieve by the method proposed in [7]. Second, when the method proposed in [7] reaches an accuracy of 0.9%, the mean accuracy of ALMI is about one time the standard deviation larger than 0.9.

To test these findings about improving accuracy for statistical significance, we apply two tests in order to check whether the variance and/or the mean of the accuracy obtained after 279 iterations differs significantly depending on whether ALMI or the reference method from [7] is used. For this, let $A=(a_{LV}^{279,1},\ldots ,a_{LV}^{279,200})$ denote the accuracy values obtained by ALMI after 279 iterations and let $B=(b_{LV}^{279,1},\ldots ,b_{LV}^{279,200})$ denote the according accuracy values obtained by the method proposed in [7]. As a first test, we use the Levene test [29] to test if the variances of *A* and *B* differ significantly. In the Levene test, the null hypothesis states that the variances of *A* and *B* are equal. Next, a *p*-value is computed that describes the probability that the variances of *A* and *B* differ more or equal than the actually observed variances under the assumption of the null hypotheses. In our experiment, the Levene test results in a *p*-value of about $3\times 10^{-9}$. This is by far smaller than the typically chosen threshold of 0.05 which shows that the null hypothesis should be rejected and the difference of variances is highly significant. As a second test, we apply a one-sided Welch’s test [30] to test if the mean of *A* is significantly larger than the mean of *B*. In the one-sided Welch’s test, the null hypothesis states that the mean of *A* is lower or equal than the mean of *B*. Next, a *p*-value is computed that describes the probability that mean(A)−mean(B) is larger or equal than the actually observed difference between the means of *A* and *B* under the assumption of the null hypothesis. In our experiment, the Levene test results in a *p*-value of about $3\times 10^{-12}$. This is by far smaller than the typically chosen threshold of 0.05 which shows that the null hypothesis should be rejected and the difference of the accuracies is highly significant.

## 5. Discussion and Conclusions

The aim of this work was to present a generic method for marine image classification that shows an improved learning performance due to the use of generic features and a reasoned choice of training samples in order to increase the efficiency of the manual annotation task performed by human experts. The proposed method ALMI is a single label-image classification method, i.e., the images of the processed dataset are required to contain maximum one object per image. However, if that is not the case, single-object images can be extracted from large scale images prior to using ALMI fully automatic. Some methods are proposed in [14,15,16] where the method proposed in [16] expects images from fixed cameras. The other two methods can be applied to any kind of dataset, e.g., image from OFOSs, FUOs, or semi-mobile platforms such as pan/tilt units.

To evaluate the extent to which the method meets this objective, its performance was compared to other related works. The evaluation focused on several aspects. First, the method was evaluated on very disparate data sets in order to assess the effectivity of the generic feature approach. Second, results from previously published evaluations of existing methods on the same data sets had to be available so results can be reproduced. Third, the evaluation was done in the same way as the previously published evaluations in order to visualize the progress. Regarding the evaluation in Section 4.2, one may observe that the data was not split into test set and training set, which is of course common practice. However, in active learning, it makes sense to leave the training data in the test set to avoid a decrease of the test set’s quality. The decrease of the test set’s quality is more prominent in a setup where object classes are underrepresented, and the true positive non-object samples are not considered in the accuracy: As discussed above, an important feature of a good sampling strategy is to draw samples from (potentially underrepresented) classes that contain actual objects of interest. If these samples are removed from the test set, the test set’s quality decreases faster with a “good” sampling strategy than with a strategy that draws many “no object” samples. This is especially illustrated by the following two points, which become only apparent when the training data are removed from the test set and become apparent more quickly with a good sampling strategy than with a sampling strategy that selects many “no object” samples.

1. When all the samples of an underrepresented class are in the training set, the underrepresented class is not part of the test set anymore.
2. When all the samples that are not in the “no object” class are in the training set, the accuracy is 0 because the test set only contains “no object” images that are not counted as true positives.

In our experiments on two data sets that use the same evaluation and the same data set selected by the authors of the previously published method, ALMI shows that

1. it can achieve higher accuracies than previously published methods and
2. it has a steeper learning curve than, i.e., ALMI achieves a certain level of accuracy with less training samples.

These effects are more prominent on the data set HG and are especially remarkable on the data set LV, because on this data set the previously published method outperformed the results of other known methods to such an extent that further improvement seemed difficult to achieve. Considering the large differences between the two marine image data sets, this all suggests that ALMI has the potential to apply to a wide range of marine image data sets and makes us confident that it can be useful in the biodiversity estimation in different types of marine habitats.

## Data Availability

Links to the orignal Hausgarten images described in Section 2 paragraph 1 can be downloaded at https://doi.pangaea.de/10.1594/PANGAEA.615724?format=textfile (Last accessed on 30 December 2020). The original LoVe images described in Section 2 paragraph 2 are available under https://love.equinor.com/ (Last accessed on 30 December 2020).
